# Differentiating objective and subjective dimensions of social isolation and apprasing their relations with physical and mental health in italian older adults

**DOI:** 10.1186/s12877-020-01864-6

**Published:** 2020-11-16

**Authors:** Maddalena Fiordelli, Gabriele Sak, Benedetta Guggiari, Peter J. Schulz, Serena Petrocchi

**Affiliations:** 1 Institute of Communication and Health (ICH), Università della Svizzera italiana (USI), Via G. Buffi 13, 6900 Lugano, Switzerland; 2 Faculty of Psychology, Università Cattolica del Sacro Cuore, Via Gemelli 1, 20123 Milan, Italy

**Keywords:** Social isolation, Loneliness, Older adults, Validation, Physical health, Mental health, Italian translation, Italian validation

## Abstract

**Background:**

International research shows that social isolation is harmful for health, especially for the elderly. Its objective and subjective dimensions are important to distinguish as each stands in a different relation with health. The first aim of the present study is the validation of three scales measuring objective and subjective isolation in an Italian elderly population. The second aim is to analyze subjective and objective social isolation and to appraise their association with health among seniors.

**Methods:**

This cross-sectional survey collected data from 306 over 65 s participants. Questionnaires were administered face-to-face by one author and encompassed: social disconnectedness scale; perceived isolation scale; abbreviated Lubben Social Network Scale; measures of general and mental health, and depression.

**Results:**

The three scales measuring social isolation demonstrated acceptable psychometric properties and validity. Objective and subjective social isolation were not directly associated with physical health, whereas subjective isolation is strongly linked to worse mental health and depression. Higher level of subjective isolation was associated with lower level of physical health through the mediation of mental health. Subjective isolation served as a mediator in the relation between objective isolation and health. Moderation analysis demonstrated that low values of objective isolation predicted high values of mental health but only when subjective isolation was low. None of these relations were moderated by socio-demographic variables.

**Conclusion:**

Subjective and objective isolation are clearly two separate dimensions and the scales validated in this paper showed to be potentially culturally invariant. Researchers should work to find instruments able to depict the complexity of the construct of social isolation.

**Supplementary Information:**

The online version contains supplementary material available at 10.1186/s12877-020-01864-6.

## Background

Western cultures are experiencing social and demographic trends that pose many novel individual, community and societal challenges. Among these issues, social isolation appears particularly relevant as it is proven to have detrimental effects on both physical and mental health [1–3]. Older adults are particularly subject to the risk of social isolation [1, 4]. Italy is the second country in the world in terms of the oldest population [5]. In their cross-national study, Fokkema et al. [6] showed higher rates of loneliness in Italy, Greece and Spain as compared to Denmark, Switzerland and the Netherlands.

Social isolation has many definitions [7] spanning from mere mentions of the absence of contacts to more complex constructs composed of two or more dimensions. Hawton et al. describe social isolation as the absence of contact with other people [8] and Berkman [9] as the “irreversible loss of social attachment and community ties” and LaVeist et al. [10] as “lack of interaction of contact with individuals within one’s social network”. More complex definitions encompass several dimensions putting the accent on feelings connected with the absence of contacts, such as support, belongingness, fulfillment and engagement. This is the case of Nicholson’s definition of isolation as “a state in which the individual lacks a sense of belonging socially, lacks engagement with others, has a minimal number of social contacts and they are deficient in fulfilling and quality relationships” [11].

In-between the simple and the complex definitions, many authors underline the bi-dimensional nature of the construct [2, 12], by distinguishing an objective and a subjective component of social isolation. For Cacioppo and his colleagues [13], for instance, social isolation is a complex construct constituted by the objective component of social disconnectedness and the subjective component of self-perceived isolation [12]. Interestingly enough, the objective dimension, such as the size of a person’s social network, does not correlate with the subjective dimension, “defined by loneliness and a perceived lack of social support” [12], or the correlation is low [14]. This means that persons with fewer social contacts do not necessarily feel lonely or isolated, while having many social contacts does not preclude a sense of isolation [15, 16]. It is of primary importance to distinguish the objective and subjective dimensions when measuring social isolation.

It is possible to retrieve numerous indicators of social isolation, developed by different disciplines and covering the different definitions above mentioned. Among the most solicited and best validated measures of subjective social isolation there are (1) the UCLA Loneliness Scale [17] and (2) the De Jong Grieveld Loneliness Scale [18, 19]. The first one is a 20-item scale which has been revised in 1996 from its original version of 1978, and it has been largely used to measure quality of relationships in adults. The De Jong Grieveld Loneliness Scale is an 11-item scale that has been developed and extensively tested in Europe and beyond. It covers two aspects of perceived isolation: emotional and social loneliness. To cover the objective component of social isolation, researchers created measures to appraise structural aspects of one’s social network [20]. Indeed, according to the AARP Foundation [21], the most popular measure used in both practice and research settings is the abbreviated Lubben Social Network Scale (LSNS-6) [20]. In an attempt to capture the global conceptualization of the construct, Cornwell & Waite [12, 16] developed a measure to evaluate social isolation called: the social disconnectedness and the perceived isolation sub-scales.

Objective and subjective isolation measures are often applied without the other, which precludes complete appraisal of social isolation, missing the exact linkages between the two dimensions, as well as their separate relations with both mental and physical health [12].

### Social isolation and health

Social isolation is not just a threat for quality of life per se, but also (and primarily) for health both for adults [2] and seniors [2, 7, 22]. The magnitude of health risks associated with social isolation is comparable with that of cigarette smoking and other major risk factors [23].

Both objective and subjective isolation showed to impact health, but on different pathways [2]. The objective dimension is linked more strongly to physical health because it may hinder healthy behaviors leading to better health outcomes [24–26]. Several authors underline that stressful relationships with one’s network can also lead to worse health outcomes [27, 28]. On the other side, the subjective dimension is found to be linked more strongly to mental health and depression [16] than to healthy behaviors and physical health [29–31]. With the words of Cacioppo & Cacioppo [2] “the extent to which an individual feels socially isolated (i.e., loneliness) predicts not only morbidity and mortality but also several specific deleterious physiological processes above and beyond what can be predicted by objective isolation”. The association between isolation and physical and mental health could be through an indirect link [16]. Objective isolation may represent a risk for health outcomes only when it results in higher subjective isolation which, in turn, leads to worse health outcomes because of the principle that subjective isolation increases stress and decreases self-efficacy [32, 33]. A systematic review has found that subjective isolation contributes to decreasing individuals’ mental health and, specifically, worsens sleep disturbance and depression [34]. Furthermore, such behavioral symptoms may have a negative impact on individuals’ general functioning, quality of life, and physical health [35].

The distinction among objective and subjective isolation can help to disentangle the different relations among constructs and to appreciate the specific role that each component of social isolation plays for mental and physical well-being [16]. It is thus essential to assess both dimensions of social isolation, and to have validated measures that can be used to this extent. Under these premises we built the current study, whose objectives are stated in the following.

### Purpose of the study

The first aim of the present study is the validation of the Italian version of the Lubben Social Network Scale short version [20] and the Social Disconnectedness Scale by Cornwell & Waite [12, 16], as well as of the Perceived Isolation Scale by Cornwell & Waite [12, 16]. We therefore expected to find acceptable internal consistency and to replicate the expected two-factor structure for all three scales (i.e., structural validity). The construct or convergent validity were tested through correlations. Although it was expected to find significant correlations among all scales, the one between the Social Disconnectedness Scale and the LSNS-6 would be expected to be strongest because they both cover the same (objective) dimension of the social isolation. Concurrent and discriminant validity were also tested through correlations with measures of health (i.e., mental and physical health, and depression).

The present research also aimed to evaluate the two components of social isolation in a senior population. To this extent, we tested four hypotheses. It was expected that objective and subjective dimensions of social isolation would be correlated weakly or moderately (*Hypothesis 1*).

The second aim of the research was to analyze the associations between objective and subjective social isolation and physical and mental health. It was expected that the objective dimension of social isolation would be associated with physical health, whereas the subjective dimension would be associated with mental health (*Hypothesis 2*). It was expected that the association between objective isolation and physical and mental health would be mediated by subjective isolation (*Hypothesis 3*). Subjective isolation could be also linked to physical health through the mediation of mental health (*Hypothesis 4).* Another possible way of interaction between social isolation and health outcomes could be multiplying. In this vein, the relationship between objective isolation and mental and physical health could be moderated by subjective isolation. It was explored whether size, sign, and strength of the relation between objective isolation and mental and physical health changed under the moderation of subjective isolation (*Research Question 1*). To deepen further the relationships between social isolation and health, the present research explored also the potential role of socio-demographic characteristics as moderators in the relations mentioned (*Research Question 2*).

## Methods

### Study participants

The sample was composed of 306 Italian people aged 65 or over. The main socio-demographic features of the sample are shown in Table 1.

**Table 1:** Participant characteristics (*N* = 306) and results from the t-tests (t) and Mann-Whitney tests (U)

Variables	N (%)	SD	PI	LSsw	Physical Health M (sd), t-test	Mental Health M (sd), t-test	Depression M (sd), t-test
**Age**							
65–74	148 (48.4)	–	–	–	–	–	–
75–84	108 (35.3)						
85 or over	50 (16.3)						
**Sex**							
Male	132 (43.1)	−0.06 (.41)	−0.05 (.59)	16.37 (6.63)	2.98 (.95)	3.17 (.91)	12.18 (4.28)
Female	174 (56.9)	0.08 (.43) t (304) = −2.78**	0.05 (.54) t (304) = −1.76	15.48 (5.99) t (288) = 1.19	2.51 (.92) t(303) = 4.44***	2.60 (.86) t(303) = 5.61***	14.83 (4.43) t(297) = − 5.19***
**Marital Status**							
Married or living common law	188 (61.5)	0.22 (.46)	−0.08 (.49)	13.87 (6.4)	2.63 (1.02)	3.03 (.92)	12.41 (3.69)
Widowed, separated or divorced, single (never married)	118 (38.5)	−0.10 (.35) t (202.74) = 6.60***	0.16 (.65) t(198.707) = 3.52**	17.09 (5.87) t (288) = −4.38***	2.76 (.92) t (303) = −1.13	2.56 (.87) t(303) = − 4.36***	15.77 (5.06) t(184.283) = 6.14***
**Background Origin**							
Italian	299 (97.7)	-up	–	–	–	–	–
Other Origin	7 (2.3)						
**Educational Attainment**							
Low (No diploma or elementary or Middle school)	161 (52.6)	0.20 (.59)	0.05 (.62)	14.71 (6.09)	2.71 (1)	2.81 (.97)	1.85 (.65)
High (High school or University)	145 (47.4)	−0.11 (.53) t(304) = 4.84***	−0.05 (.49) t (304) = 1.67	17.27 (6.23) t (288) = −3.53**	2.71 (.89) t (303) = −.029	2.90 (.86) t (303) = −.81	1.75 (.52) t (303.9) = 1.46
**Religion Affiliation**							
Cshristian	263 (85.9)	–	–	–	–	–	–
Atheist or agnostic	20 (6.5)						
Refused to answer	23 (7.7)						
**Religious Engagement**							
No	247 (91.2)	0.003	−0.24	16	3	3	1.62
Yes	24 (8.8)	−0.04 U = 5352.5	−0.14 U = 6255.5	16 U = 5040	3 U = 4518	3 U = 4243.5	1.68 U = 5952

*SD* Social Disconnectedness, *PI* Perceived Isolation, *LS* Lubben Scale

* *p* < .05; ** *p* < .01; *** *p* < .001

Independent t-test showed significant differences by sex on social disconnectedness, physical and mental health. Moreover, the seniors without a partner perceived themselves to be more isolated than those who had a spouse/partner for both subjective and objective isolation. Differences emerged also on mental health and depression. Considering the educational level, participants with high educational level showed lower scores on the dimension of isolation than participants with low educational level. Analysis on religiously engaged did not yield significant results.

### Procedure

Participants were recruited through snowball sampling and data collection took place between September 2016 and April 2017 in the North-Italian area. The North-Italian area was chosen because elderly are more likely to live in big urban or suburban areas and this create an interesting case study for studying the relationships among social isolation, loneliness, and health. The information sheet of the research was advertised to possible participants through the collaborations with voluntary associations, meeting groups for elderly, veteran groups, labor unions, universities of the third age, and rest homes. In light of the advanced age of the sample, the survey was developed as a paper-pencil questionnaire and administered as a face-to-face interview in different locations (e.g., participants’ private homes, older adults’ retirement houses, public locations, and recreational centers). Participant recruitment and data collection took place in the North-Italian area. The survey was administered face-to-face in participants’ private homes, older adults’ retirement houses, public locations, and recreational centers. The sample was stratified by age, sex, marital status, living situation, and educational attainment according to the demographic characteristics of the Italian senior population (ISTAT, 2015–2016). Inclusion criteria were: 1) being 65 years old or over, 2) speaking Italian, 3) possess sufficient cognitive ability to answer the questions autonomously.

The two scales measuring social isolation were translated into Italian by a native speaker and back-translated into English by a bilingual speaker. The instruments were pretested with 20 subjects who found the questions clear and understandable. The researcher provided information about the purpose of the study to the participants and the informed consent was signed by each of them. The average duration of the interview was 20 min.

### Measures

#### Objective isolation

The social disconnectedness scale [12] includes a set of five items assessing social network size and a set of three items measuring social activity. The scores were reverse-coded, standardized, and averaged to obtain a measure of disconnectedness [12, 16].

The LSNS-6 [20] is a six-item scale assessing one’s integration with family and friends. The responses are summed up to gain a composite score ranging from 0 (very limited social network) to 30 (very large social network), where 12 is considered to be the cut-off for disconnectedness [20].

#### Subjective isolation

The perceived isolation scale [16] integrates a set of three items assessing loneliness, and a set of six items evaluating perceived social support. The two scores were reverse-coded, standardized and averaged to obtain a subjective measure of perceived isolation.

#### Physical and mental health

Self-rated physical and mental health were appraised through one item per each. Depressive symptoms were assessed through the shortened measure developed by the Center for Epidemiological Studies Depression Scale (CES-D) [36]. Participants were prompted to provide the estimated frequency (ranging from 1 = “rarely or never”, to 4 = “most of the times”) they experienced ten different moods in the past week (e.g., “I did not feel like eating: my appetite was poor”, “I was happy”, “I felt that people disliked me”). The final score was averaged with higher score indicating higher depressive symptoms.

### Statistical procedure

Data were analyzed with the SPSS 23 and AMOS 24. We calculated the internal consistency applying Cronbach’s alpha to evaluate the reliability of the scales which were also subjected to confirmatory factor analyses (CFA) using Structural Equation Modelling (SEM). The SEM models tested the two factor-structure. After estimating the expected solution, the modification indices were observed to improve the model. The largest covariance between errors tapping the same factor was added to the model. We used the χ^*2*^-value, the CFI (Comparative Fit Index), and the RMSEA (Root Mean Error of Approximation) in order to estimate the fit between the data and the model. The χ^*2*^ values should be non-significant, the CFI should be greater than or equal to 0.95, and the RMSEA lower than .08 [37].

Individual t-tests were calculated to test significant differences by sex, education, or marital status on social isolation measures, physical and mental health, and depression. Non-parametric tests were carried out with religious engagement. Correlations and Hierarchical Regression Analyses (HRA) were carried out to test the expected relationships between variables. In the HRAs, the predictors were: sex, education, marital status, and religious engagement dummy coded (Step 1), objective isolation (i.e. Social Disconnectedness or Lubben scale, alternately inserted in the regression in order to avoid multicollinearity; Step 2), and subjective isolation (i.e., Perceived Isolation; Step 3). Outcomes were self-rated physical and mental health and depression.

Mediation analysis was applied to determine whether subjective isolation served as a mediator in the relation between objective isolation and health and whether the relation between subjective isolation and physical health was mediated by mental health.

To deepen further the relationships between social isolation and health, the present research explored the potential role of socio-demographic characteristics as moderators in the relation. Mediation and moderation analyses were carried out with Process 2.15 macro for SPSS as suggested by Preacher & Hayes [38]. To further probe the interaction the Johnson-Neymar technique was applied [39].

## Results

### Psychometric properties and CFA of the scales

The Social Disconnectedness Scale yielded a Cronbach’s alpha of .64 indicating moderate internal consistency. The item-test correlations exceeded the value of .27 indicating satisfactory reliability [40], with the exception of item 6 and item 7. The Perceived Isolation Scale attained a Cronbach’s alpha of .68 and item-test correlations > .25. The CES-D scale demonstrated acceptable internal consistency (α = .70) after the exclusion of item 6 and item 10, and item-test correlations > .20. The internal consistency of the LSNS-6 was .82 and item-test correlations were > .41).

**Fig. 1:**
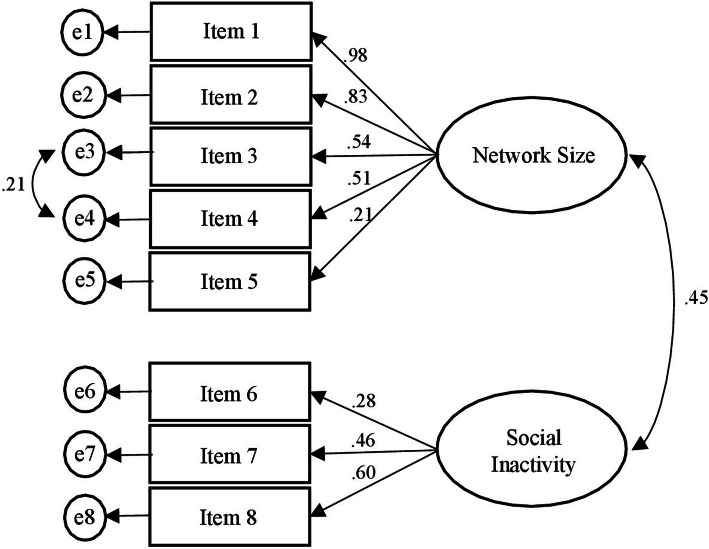
Confirmatory Factor Analysis for the Social Disconnectedness scale

According to Cornwell & Waite [11], the CFA on the 8 Social Disconnectedness items tested the expected two-factor structure (i.e., network size – 5 items - and social inactivity – 3 items). The model showed a χ^*2*^ (19) = 53.59, *p* < .0001. Although significant, this statistic should be used with caution because it is inflated by the large sample size. The CFI = .93 and the RMSEA = .07 (LO90 = .05; HI90 = .10) showed a moderate to good fit of the data. See Fig. 1 for details.

**Fig. 2:**
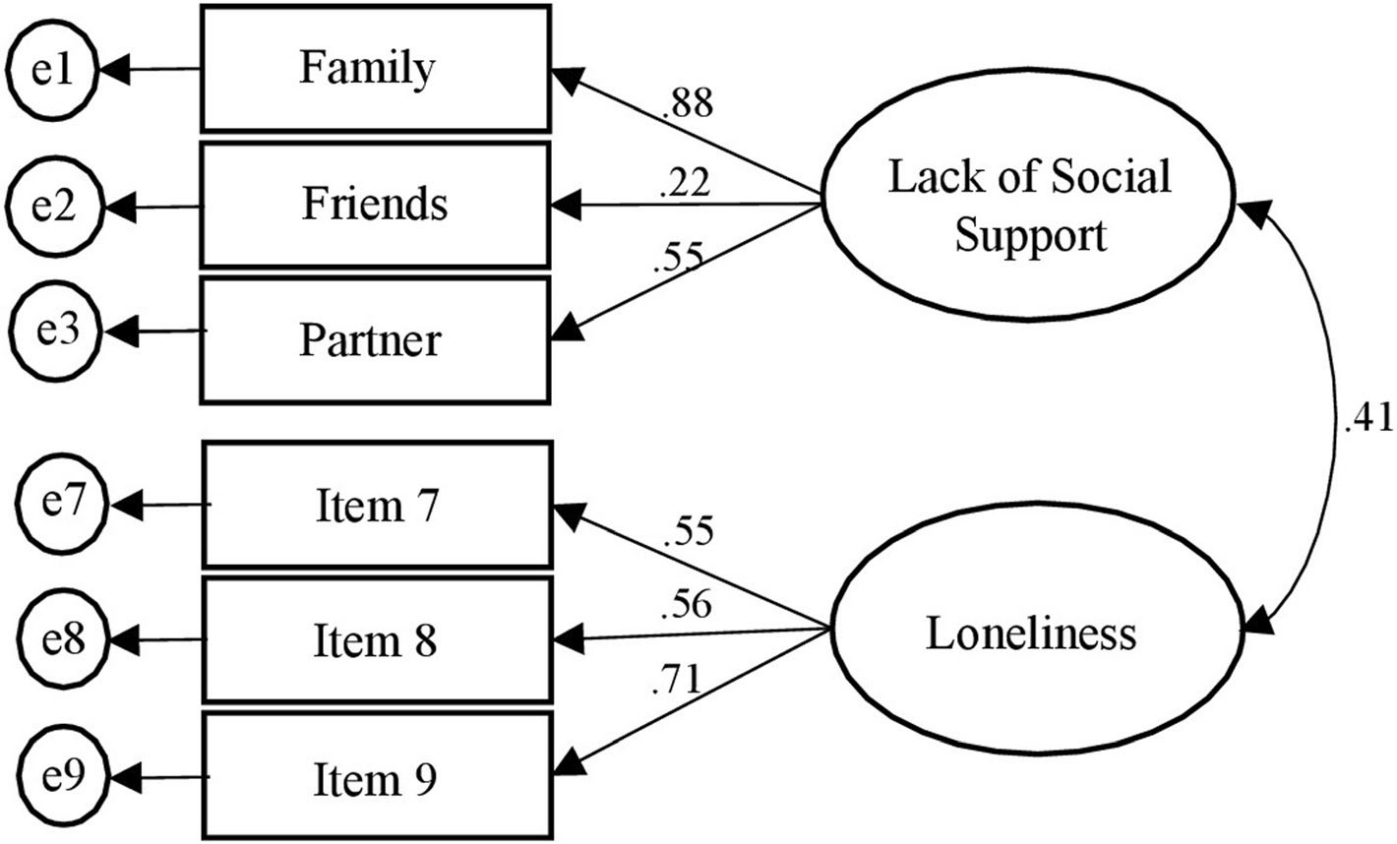
Confirmatory Factor Analysis for the Perceived Isolation scale

According to Cornwell & Waite [16] two factors were estimated through a CFA from the 9 items of subjective isolation, i.e., loneliness and lack of social support. The analysis yielded inadequate goodness of fit, χ2 (26) = 196.07, *p* < .0001, CFI = .65, RMSEA = .15. The errors of the 6 items of the lack of social support were correlated two by two (i.e., errors of items on family/or friends/ or partner correlated to each other). Thus, a second CFA was performed estimating two factors, the first on the 3 items of the lack of social support (from family, friends, and partner) calculated as the average of the original 6 items and the second factor on the 3 items of loneliness. The CFA showed good fit of the data, χ2 (8) = 12.31, *p* = .14, CFI = .98, and RMSEA = .042, (LO90 = .000, HI90 = .08). See Fig. 2 for details of the second CFA.

**Fig. 3:**
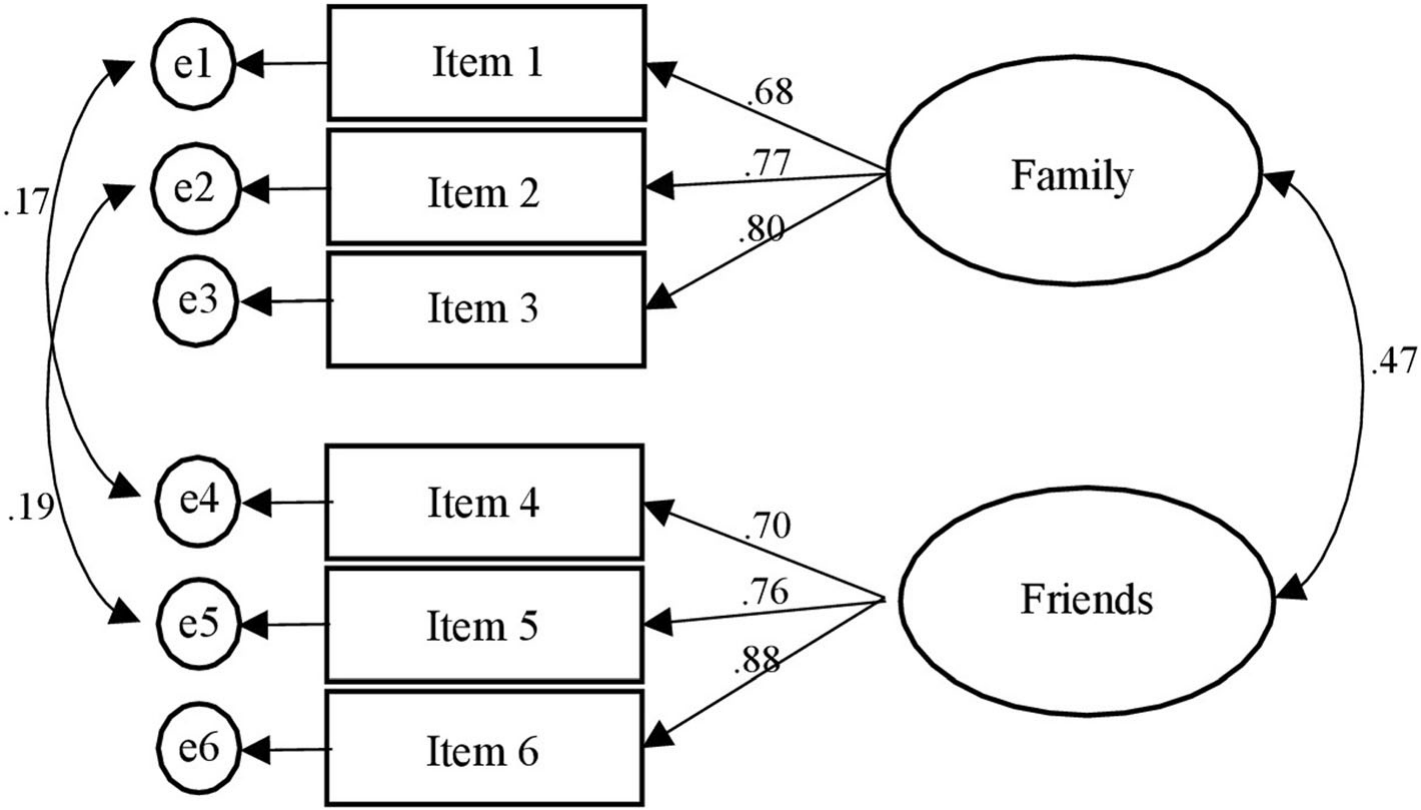
Confirmatory Factor Analysis for the Lubben scale

The LSNS-6 was checked with SEM and the expected two-factor structure emerged*,* χ2 (6) = 17,389, *p* = .006, CFI = .98, RMSEA = .06, (LO90 = .039, HI90 = .12). See Fig. 3 for details. According to Lubben et al., [15] the cutoff for social isolated people identified 25.2% of the participants with a score lower than 12.

### Relationships among measures

The three scales correlated each other (see Table 2) es expected. Other correlations emerged between Social Disconnectedness and Perceived Isolation and depression (positive), Perceived Isolation, physical (negative) and mental health (negative), the LSNS-6 and depression (negative). Physical and mental health correlated with each other (positive) and with depression (negative). Age correlated with subjective and objective isolation, and depression. Sex was significantly correlated with social disconnectedness and physical and mental health. Social Disconnectedness and Perceived Isolation were moderately correlated with each other, Perceived Isolation was weakly correlated with the LSNS-6, whereas Social Disconnectedness was highly correlated with the LSNS-6.

**Table 2:** Correlations between measures

	*M* (*SD, range*)	SD	PI	LS	PH	MH	D
Age	76.11 (7.5, 65–96)	.36***	.21***	−.25***	−.11	−.07	.24***
Sex		.16**	.10	−.07	−.25***	−.31***	.30***
Social Disconnectedness (SD)	.018 (.43; −.89–1.79)		.34***	−.58***	−.07	−.11	.27***
Perceived Isolation (PI)	.0068 (.56; −.60–2.65)			−.29***	−.15**	−.22***	.44***
LSNS-6 (LS)	15.86 (6.2; 0–30)				.08	.03	−.20**
Physical Health (PH)	2.71 (.96; 1–5)					.39***	−.33***
Mental Health (MH)	2.85(.93; 1–5)						−.48***
Depression (D)	1.7 (.56; 1–4)						

* *p <* .05; ** *p <* .01; *** *p <* .001. 287 < df < 304

In both HRAs with physical health as outcome, sex was the only significant predictor (t < − 3.66, *p* < .0001) with women showing less physical health than men do (*β* = −.25 for both models). Neither objective isolation nor subjective isolation were significant predictors of physical health. Tables 3 and 4 show results of the HRAs respectively with mental health and depression mood as outcomes.

**Table 3:** Non-automatic hierarchical regression analysis with mental health as dependent variable

	Step 1			Step 2			Step 3		
	B	SE(B)	*β*	B	SE(B)	*β*	B	SE(B)	*β*
Age	−.004	.008	−.03	−.004	.008	−.03	−.003	.008	−.02
Sex (0 = male)	−.37	.12	−.20**	−.37	.12	−.20**	−.38	.12	−.20**
Education (0 = low)	−.009	.11	−.005	−.02	.11	−.008	−.03	.11	−.01
Marital Status (0 = no partner)	.25	.12	.13*	.24	.13	.13	−19	.13	.10
Religious engagement (0 = no)	−.25	.14	−.10	−.25	.15	−.11	−.23	.14	−.10
Social Disconnectedness (Lubben Scale)				−.035 (−.005)	.14 (.009)	−.02 (−.04)	.08 (−.01)	.15 (.009)	.04 (−.07)
Perceived isolation							−.29 (−.27)	.10 (.10)	−.18** (−.17**)
R^2^ (R^2^ adj.)	.11 (.09)			.11 (.09)			.14 (.11)		
F for change in R^2^	(5264) = 6.35***			(1263) = .06			(1262) = 8.46**		
F	(5264) = 6.35***			(6263) = 4.12***			(7262) = 5.87***		

Between brackets results from the HRA when the Lubben scale was considered as independent variable

* *p* < .05; ** *p* < .01; *** *p* < .001. 287 < df < 304

**Table 4:** Non-automatic hierarchical regression analysis with mood as dependent variable depression

	Step 1			Step 2			Step 3		
	B	SE(B)	*β*	B	SE(B)	*β*	B	SE(B)	*β*
Age	.012	.005	.15*	.011	.005	.13	.01	.005	.12*
Sex (0 = male)	.26	.08	.22**	.26	.08	.22**	.27	.07	.22***
Education (0 = low)	−.08	.07	−.06	−.06	.07	−.05	−.04	.07	−.03
Marital Status (0 = no partner)	−.28	.08	−.23***	−.27	.08	−.22**	−.18	.08	−.14*
Religious engagement (0 = no)	−.08	.09	−.05	−.08	.10	−.05	−.10	.09	−.07
Social Disconnectedness (Lubben Scale)				.14 (−.007)	.09 (.006)	.10 (−.07)	−.01 (.001)	.09 (.006)	−.009 (.01)
Perceived isolation							.39 (.36)	.06 (.06)	.37*** (.36***)
R^2^ (R^2^ adj.)	.17 (.15)			.17 (.16)			.29 (.28)		
F for change in R^2^	(5265) = 10.61***			(1264) = .13			(1263) = 44.54***		
F	(5265) = 10.62***			(6264) = 9.28***			(7263) = 15.63***		

Between brackets results from the HRA when the Lubben scale was considered as independent variable

* *p <* .05; ** *p <* .01; *** *p <* .001. 287 < df < 304

Women and participants with high perceived isolation scores showed low scores on their mental health and high scores on their depression mood. Age and not having a partner were significant predictors of depression mood.

It was tested whether subjective isolation served as a mediator between objective isolation and health. Table 5 shows the results. The findings yielded support for the full mediation between objective isolation, physical health, mental health, and depression (when the LSNS-6 was considered). Objective isolation was a significant predictor of subjective isolation, which was associated with physical and mental health and depression. There was also a partial mediation between social disconnectedness and depression. In this case, social disconnectedness was a significant predictor of both perceived isolation and depression and perceived isolation predicted an increase in depression.

**Table 5:** Mediation analyses between objective isolation (X), health outcomes (Y) under the moderation of subjective isolation (M)

Antecedent	Consequent																	
	M (Perceived Isolation)			Y (Physical Health)			M (Perceived Isolation)			Y (Mental Health)			M (Perceived Isolation)			Y (Depression)		
	Coeff [LLCI ULCI]	*SE*	*t*	Coeff [LLCI ULCI]	*SE*	*t*	Coeff [LLCI ULCI]	*SE*	*t*	Coeff [LLCI ULCI]	*SE*	*t*	Coeff	*SE*	*t*	Coeff	*SE*	t
X (Social Disconnectedness)	0.45 [.30 .58]	0.07	6.29***	− 0.06 [−.32 .20]	0.13	−.46	0.43 [.29 .57]	0.07	6.14***	− 0.08 [−.3 .17]	.12	−.61	0.44	0.7	6.14***	1.61	0.57	2.78*
M (Perceived Isolation)	–	–	–	− 0.23 [−.43 -.03]	0.10	−2.33*	–	–	–	− 0.34 [−.53 -.14]	0.09	−3.48**	–	–	–	3.13	.43	7.24***
constant	0.0008	0.03	.025	2.71	0.05	49.72***	−0.004	0.03	−.13	2.85	0.05	54.89***	0	0.03	0	13.64	0.23	7.23***
	R^2^ = .12 F (1 303) = 39.59***			R^2^ = .02 F (2 302) = 3.61*			R^2^ = .11 F (1 303) = 37.76***			R^2^ = .05 F (2 302) = 7.82**			R^2^ = .11 F (1 297) = 37.76***			R^2^ = .22 F (2 296) = 41.54***		
X (Lubben Scale)	− 0.03 [−.03 -.01]	0.01	−5.08***	0.01 [−.01 .02]	0.01	.84	− 0.02 [−.03 -.015]	0.005	−4.92***	− 0.004 [−.02 .013]	0.01	−.45	−0.2	0.005	−4.99***	−0.06	0.04	−1.56
M (Perceived Isolation)	–	–	–	−0.21 [−.41 -.009]	0.10	−2.05*	–	–	–	− 0.34 [−.53 -.15]	0.09	−3.54**	–	–	–	3.33	0.44	7.48***
constant	−0.2	0.01	−5.08***	2.59	0.15	16.31***	0.41	0.08	4.64***	2.91	0.15	19.35***	0.42	0.08	4.75***	14.65	.69	21.01***
	R^2^ = .08 F (1 288) = 25.85***			R^2^ = .02 F (2 287) = 3.23*			R^2^ = .08 F (1 287) = 24.20***			R^2^ = .04 F (2 286) = 6.42*			R^2^ = .08 F (1 282) = 24.95***			R^2^ = .20 F (2 281) = 35.45***		

* *p* < .05; ** *p* < .01; *** *p* < .001. 287 < df < 304

Mental health and depression should serve as a mediator between subjective isolation and physical health. When depression was the mediator, there was a significant effect of subjective isolation on depression (β = 3.56, SE = .40, *p* < .001, LLCI = 1.95, ULCI = 4.22) and depression on physical health (β = −.07, SE = .02, *p <* .001, LLCI = -.09, ULCI = -.05). When perceived mental health was the mediator, there was a significant effect of subjective isolation on mental health (β = −.37, SE = .04, *p* < .001, LLCI = -.48 -.0003) and mental health on physical health (β = .39, SE = .05, *p <* .001, LLCI = .30, ULCI = .51). In both mediation analysis, the direct effects were not significant.

**Fig. 4:**
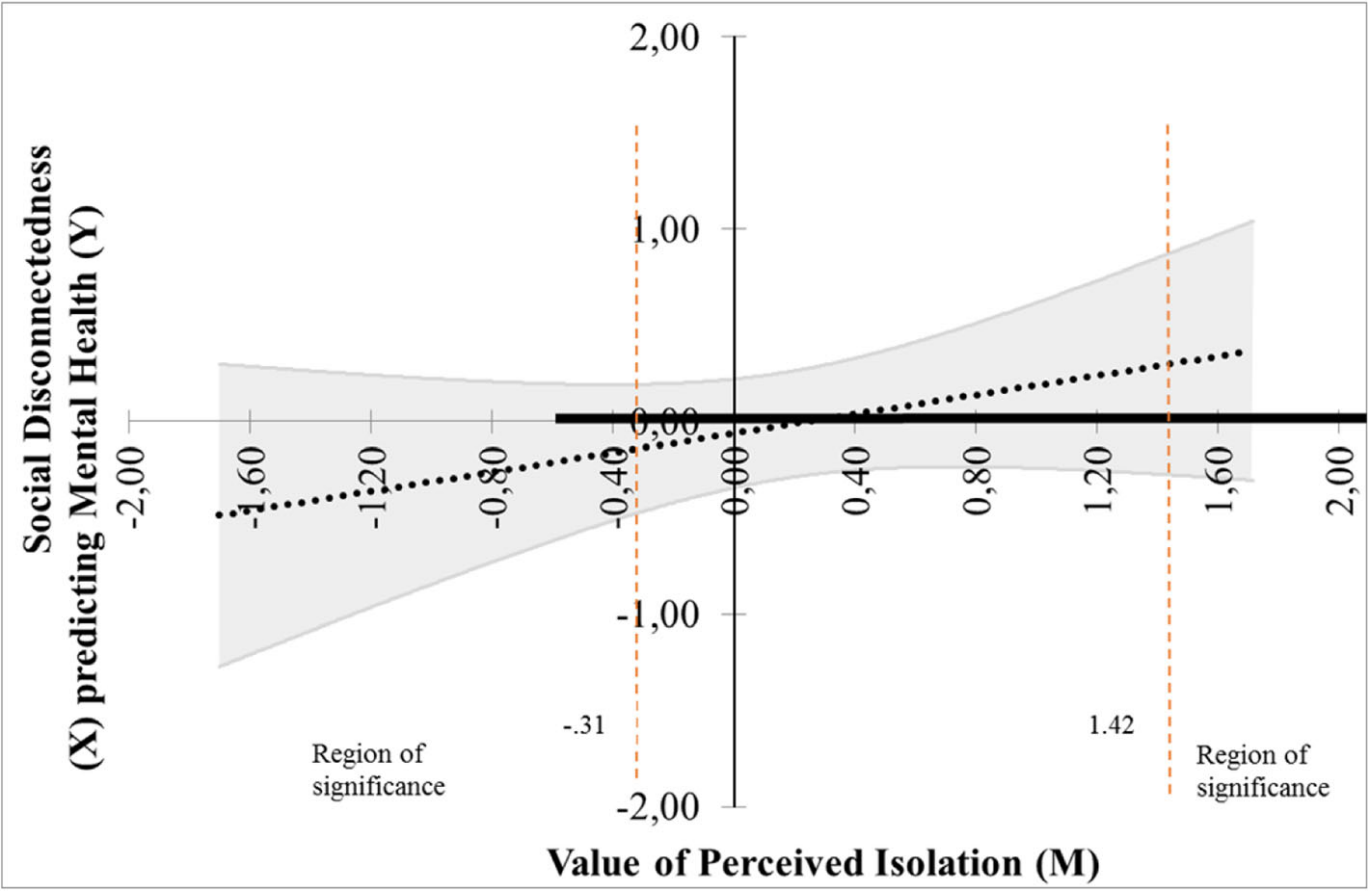
Probing interaction with the Johnson-Neymar technique for the social disconnectedness predictor and perceived isolation moderator

The findings yielded support for the moderation effect of the perceived isolation on the relation between social disconnectedness and mental health, F(3 301) = 7.89, *p* < .001. The interaction was significant, t(301) = 2.71, *P* = .007, β = .40 (SE = .15), and the main effect of perceived isolation as well, t(301) = − 4.26, *p <* .001, β = −.43 (SE = .10). The main effect of social disconnectedness was not significant. The Johnson-Neymar technique revealed two regions of significance defined by a lower bound of −.30 and an upper bound of 1.61. As shown in Fig. 4, this region implies that the regression of mental health on social disconnectedness is significant and negative at values of perceived isolation less than −.30 (corresponding to 104 of the 306 participants, 34%), not significantly different from zero at values of perceived isolation between −.30 and 1.61, and significant and positive at values of perceived isolation greater than 1.60 (corresponding to 5 participants, 1.63%). Given that the minimum and maximum values of the perceived isolation were − .60 and 2.65, respectively, both the upper and the lower region fell within the observed range of perceived isolation. However, given the fact that only 1.63% of participants had a value of perceived isolation higher than 1.60, hence this result will not be interpreted further. The lower bound of the region of significance (corresponding to 34% of participants) showed that the regression between social disconnectedness and mental health is negative if the values of perceived isolation are low. In other words, low values of social disconnectedness predicted high mental health but under the effect of low level of perceived isolation.

Results from the moderation analyses showed that the relations between subjective isolation and mental health/depression are not moderated by sex, t(302) = −.42, *p* > .05, β = −.07 (SE = .17), religious engagement, t(302) = − 1.88, *p >* .05, β = −.42 (SE = .22), marital status, t(302) = −.50, *p >* .05, β = −.09 (SE = .18), and education, t(302) = −.11, *p >* .05, β = −.04 (SE = .16).

## Discussion

The general purpose of this study was testing the hypothesized connections between social isolation and worse physical health, mental health, and depression in an elderly population. A complementary purpose of this research was the validation of the Italian version of three widely adopted social isolation measures: the LSNS-6 [20], the Social Disconnectedness Scale and Perceived Isolation Scale [12, 16]. To date, there has not been an adaptation of these scales for use in Italian samples, and this study is a first attempt to do that.

Results from psychometric tests showed moderate internal consistency of the Social Disconnectedness Scale, and the Perceived Isolation Scale. As for the LSNS-6, the internal consistency was high for the Family subscale as well as for the Friends Subscale. The factorial validity of the three scales has been demonstrated. Findings from CFA showed that the two-factor structure (network size and social inactivity) of Social Disconnectedness provided the best fit to the data with the selected eight items, in accordance with the original authors of the scale. According to previous findings from the original authors, Cornwell & Waite [16], the item related to the “Lack of friends” is reflective of social inactivity rather than network size. Findings from a first CFA on the nine items of the Perceived Isolation Scale showed inadequate goodness of fit when testing the two-factor structure (lack of social support and loneliness). The errors of the six items were correlated two by two (items on: family members, friends, and spouse). This finding suggested performing a second CFA on the three items of loneliness and the three items of the lack of social support from the three distinct sources, calculated as the average of the original six items. The CFA showed good fit of the data. Our understanding of this finding is that it is the types of relationships that shape the perception of isolation of an individual rather than perceived isolation as a concept which is transversal to the three kinds of relationships. Previous studies have indeed highlighted differences of support from diverse sources [41] as well as specific impact of different sources of support on mental health in an elderly population [42]. The LSNS-6 was checked with SEM, and the expected two-factor structure of integration of family and friends emerged.

When checking for relations among the various measures we found that there was a significant difference regarding sex and the perception of physical and mental health. In line with previous evidence, men perceive a better physical and mental health status for themselves than women [43–45]. This was reflected also when measuring depressive symptoms, as women showed more symptoms than men did. The reasons for this should be further explored, but it may be that sex differences in terms of longevity play a role with respect to both perceived physical and mental health. Women living longer may experience more functional problems as well as more losses in terms of close relationships. These phenomena can respectively affect physical and mental health. The presence of more depressive symptoms in women could be explained by the differences in emotions between sexes [45]. The relationship status played a role in the perception of isolation, as adults without a partner reported more perceived isolation than the ones who have a partner, as was also shown previously by Cornwell & Waite [16]. In accordance with these findings, we found differences also for the objective measure of social isolation of the LSNS-6 and the relationship status. People without a partner had lower scores of family and friends of the LSNS-6, and the same participants showed lower scores in mental health and higher scores in depression scales as compared with individuals in a relationship.

We also found expected significant differences in the objective dimension of social isolation for people with different levels of education. Participants with a higher level of education showed significant lower objective isolation than participants with a lower educational level. Mental and physical health differ also depending on personal engagement in religion.

The Social Disconnectedness and the Perceived Isolation Scales correlate negatively with the LSNS-6 (measuring integration), while they correlate positively with depression. Perceived Isolation was negatively correlated with both physical and mental health. LSNS-6 also correlated negatively with depression. Physical and mental health correlate positively, while physical and mental health correlate negatively with depression.

As mentioned above, sex was a predictor for physical health, women showing a lower level of physical health than men [16]. However, neither objective isolation nor subjective isolation were significant predictors of physical health. Women and participants with high-perceived isolation score, showed low scores on their mental health. Age was a significant predictor of high level of depression, together with being a woman, not having a partner, and having a high score on perceived isolation. The authors of two of the scales we validated found actually that perceived isolation was a predictor for both mental health and physical health [16], perceived isolation having a stronger association than social disconnectedness with mental health. However, we could argue that this was partially due to the statistical power of their sample. In fact, in our bivariate analysis also, perceived isolation significantly correlated with mental and physical health, and the correlation was higher with mental health.

Subjective isolation served as a mediator between objective isolation and health. Mediation analyses support the link between objective isolation, physical health, mental health and depression. Moderation analyses also confirmed that perceived isolation has an effect on the relation between social disconnectedness and mental health, and this is the case when its values are low. This shows the multiplier effect of the two dimensions: low levels of disconnectedness are able to predict higher mental health but only if perceived isolation is also low. This opens up to further research, and to the need to clarify which are the relationships, and the absence of, that most affect perceived isolation. These findings could guide the development and test of interventions targeting a specific dimension of social isolation. For instance, cognitive interventions proved successful to tackle perceived isolation. Also, solitary interventions could be used to address this issue as they have proven innovative and well received from the hard to reach groups [46, 47]. In addition, objective isolation is a good predictor of subjective isolation, which is in turn associated with physical health, mental health and depression. This is in line with what the authors of the scales observe, “The relationship between social disconnectedness and mental health appears to operate through the strong association between perceived isolation and mental health. Our results suggest that if the socially disconnected older adults have worse mental health only to the extent that they feel isolated” [16].

Our results show that the two scales work well in measuring objective isolation, both in terms of integration (LSNS-6) and of social disconnectedness, in an Italian population. As regards the subjective measure of social isolation, perceived isolation, there is a suggestion for a three- factor structure (family, friends and spouse), instead of the two-factor structure found by the original authors. The Social Disconnectedness Scale, the Perceived Isolation Scale and the LSNS-6 are reliable, and their structure in the Italian population reflect the structure found by original authors. However, people from nationalities other than Italian were not represented and this is mainly because of the socio demographic structure of the nation where older adults are almost exclusively Italian. The adequacy of the scales for older adults of different nationalities living in Italy should be determined by further studies. Moreover, the sample of our study was entirely based in a northern region of Italy, and we have reasons to assume that cultural differences may be found in the central as well as in the southern regions when it comes to social isolation. Further validation studies should encompass a more heterogeneous sample in terms of culture.

Another limitation of our study concerns the sampling bias concerning the choice to recruit participants in the North-Italian area. In this part of Italy, elderly are more likely to live in big urban or suburban areas and this created a case study for subjective and objective social isolation. Data collection in other geographical areas, such as those in which elderly live in small cities with perhaps more tight connections with relatives and friends, might return a different picture about the relationships among social isolation, loneliness, and health.

## Conclusions

A gold standard to measure social isolation does not exist yet [20, 48]. Given the relevance of the topic of social isolation, its proven link with both physical and mental health, and the call from institutional bodies to investigate it further and develop effective screening measures [1, 20], it becomes essential for researchers to refine and adapt measurement tools to diverse cultural contexts. By doing so, we should not forget the importance of being able to use instruments that depict the complexity of the construct of social isolation by measuring both the objective and the subjective dimension. The combination of multiple measures for assessing social isolation therefore remains vital. As the authors of two of the scales tested in this study [16] highlight, it is important to remember that the objective and subjective dimensions of social isolation are related but distinct as this has strong implications also for designing appropriate interventions [46, 47, 49]. Having translated and tested the three scales in Italian is paramount not just for the Italian setting itself and for the importance of the country in the European landscape, but mostly because the scales have been shown to be culturally invariant, therefore highlighting their robustness and suitability for a wide assessment of a major health problem in the population [1].

## Supplementary Information

**Additional file 1.**

## Data Availability

Not applicable.

## Abbreviations

CES-D: Center for Epidemiological Studies Depression Scale
CFA: Confirmatory Factor Analyses
CFI: Comparative Fit Index
HRA: Hierarchical Regression Analysis
LSNS-6: Abbreviated Lubben Social Network Scale
RMSEA: Root Mean Error of Approximation
SEM: Structural Equation Modelling
