# Supplementary material for: Differentiating objective and subjective dimensions of social isolation and apprasing their relations with physical and mental health in italian older adults
Source: BMC Geriatr. 2020 Nov 16;20:472. doi: 10.1186/s12877-020-01864-6 (PMC7670809; doi:10.1186/s12877-020-01864-6)
Supplement: Supplementary file 1 — Additional file 1. [file 12877_2020_1864_MOESM1_ESM.docx]

**SOCIAL ISOLATION QUESTIONNAIRE**

*(Cornwell & Waite Scale and LSNS-6 scale are in their original version. We translated the other questions from Italian to add the questionnaire as supplementary material.)*

**SOCIAL DISCONNECTEDNESS SCALE (Cornwell & Waite, 2009)**

**SD1) Social network size**

From time to time, most people discuss things that are important to them with others. For example, these may include good or bad things that happen to you, problems you are having, or important concerns you may have. Looking back over the last 12 months, who are the people with whom you most often discussed things that were important to you? Please list these people in Section A of your roster.]

(PROMPT IF DON’T KNOW: This could be a person you tend to talk to about things that are important to you.)

ENTER **UP TO 5 NAMES** IN ROSTER IN THE ORDER IN WHICH THEY ARE IDENTIFIED BY RESPONDENT (SECTION A).

*PROMPT ONCE WHEN RESPONDENT IS FINISHED IF HE OR SHE HAS NAMED FEWER THAN 5 PEOPLE:

Are there any more?

**SD2) Social network range**

The following question was asked for each of the first five network members the respondent named. The resulting information was used to calculate the number of different types of relationships within one’s network.

Which of the following best describes (name)'s relationship to you? (PROMPT IF NEEDED: So this person is your…) (USE HAND CARD B)

Ex-spouse

Romantic/Sexual partner

Parent

Parent in-law

Child

Step-child

Brother or sister

Other relative of yours

Other in-law

Friend

Neighbor

Co-worker or boss

Minister, priest, or other clergy

Psychiatrist, psychologist, counselor, or therapist

Caseworker/Social worker

Housekeeper/Home health care provider

Other (Specify) _____________________

DON’T KNOW

REFUSED

**SD3) Frequency of interaction with network members**

This question was asked for each of the respondent’s five network members. To construct our measure of network contact, we recoded the categories to approximate the number of days per year that the respondent talks to each network member: less than once a year = .5, once a year = 1, a couple times a year = 2, once a month = 12, once every two weeks = 24, once a week = 52, several times a week = 182, and every day = 365. We averaged these values across all of the respondent’s network members and divided by 365. The resulting variable ranges from 0 to 1, where 0 = the respondent does not contact any alters and 1 = the respondent contacts all alters every day.

How often do you talk to this person?

*IF RESPONDENT ASKS, SAY THAT TALKING OVER THE TELEPHONE AND

PERSONAL EMAIL (I.E., EMAIL BACK AND FOR THE BETWEEN THE TWO OF YOU)

MAY BE INCLUDED.*

Every day

Several times a week

Once a week

Once every two weeks

Once a month

A couple times a year

Once a year

Less than once a year

DON’T KNOW

REFUSED

**SD4) Proportion of network alters in the home**

The following question was asked for each of the first five network members the respondent named. Note: network members were considered to be co-residents if they reside with the respondent for at least part of the year.

Does (name) live in the same household with you? (INTERVIEWER NOTE: LIVES IN SAME RESIDENCE WITH RESPONDENT, NOT IN SAME APARTMENT COMPLEX.)

Yes – lives in the same household

No – does not live in household

IF VOLUNTEERED – LIVES WITH R PART OF THE YEAR

DON’T KNOW

REFUSED

**SD5) Number of friends**

Responses were coded as 0 = “none,” 1 = “one,” 2 = “2-3”, 3 = “4-9,” 4 = “10-20,” 5 = “more than 20.”

About how many friends would you say that you have? Is that...

None

One

2-3

4-9

10-20

More than 20

DON’T KNOW

REFUSED

**SD6) Attendance at group meetings**

This question was asked in the Leave-Behind Questionnaire. (Respondents completed this after the interview and mailed it back to us.) Response categories were reverse-coded, so that they range from 1 = “never” to 7 = “several times a week.”

In the past 12 months, how often did you attend meetings of any organized group? (Examples include: a choir, a committee or board, a support group, a sports or exercise group, a hobby group, or a professional society.)

Several times a week

Every week

About once a month

Several times a year

About once or twice a year

Less than once a year

Never

**SD7) Socializing with friends and family**

This question was asked in the Leave-Behind Questionnaire. (Respondents completed this after the interview and mailed it back to us.) Response categories were reverse-coded, so that they range from 1 = “never” to 7 = “several times a week.”

In the past 12 months, how often did you get together socially with friends or relatives?

Several times a week

Every week

About once a month

Several times a year

About once or twice a year

Less than once a year

Never

**SD8) Volunteering**

This question was asked in the Leave-Behind Questionnaire. (Respondents completed this after the interview and mailed it back to us.) Response categories were reverse-coded, so that they range from 1 = “never” to 7 = “several times a week.”

In the past 12 months, how often did you do volunteer work for religious, charitable, political, health-related, or other organizations?

Several times a week

Every week

About once a month

Several times a year

About once or twice a year

Less than once a year

Never

**PERCEIVED ISOLATION SCALE (Cornwell & Waite, 2009)**

**PI1) Emotional support from family members**

When calculating the scale, responses were reverse-coded so that 1 = “often,” 2 = “some of the time,” and 3 = “hardly ever (or never).”

How often can you open up to members of your family if you need to talk about your worries? Would you say hardly ever, some of the time, or often?

HARDLY EVER (OR NEVER)

SOME OF THE TIME

OFTEN

IF VOLUNTEERED – NO FAMILY (SKIP NEXT QUESTION)

DON’T KNOW

REFUSED

**PI2) Instrumental support from family members**

This question was skipped if respondent indicated in question PI1 (above) that he/she has no family. When calculating the scale, responses were reverse-coded so that 1 = “often,” 2 = “some of the time,” and 3 = “hardly ever (or never).”

How often can you rely on them for help if you have a problem? Would you say hardly ever, some of the time, or often?

HARDLY EVER (OR NEVER)

SOME OF THE TIME

OFTEN

DON’T KNOW

REFUSED

**PI3) Emotional support from friends**

This question was not asked if respondents answered “none” to question SD5 (above). When calculating the scale, responses were reverse-coded so that 1 = “often,” 2 = “some of the time,” and 3 = “hardly ever (or never).”

How often can you open up to your friends if you need to talk about your worries? Would you say 1) hardly ever, some of the time, or often?

HARDLY EVER (OR NEVER)

SOME OF THE TIME

OFTEN

DON’T KNOW

REFUSED

**PI4) Instrumental support from friends**

This question was not asked if respondents answered “none” to question SD5 (above). When calculating the scale, responses were reverse-coded so that 1 = “often,” 2 = “some of the time,” and 3 = “hardly ever (or never).”

How often can you rely on them for help if you have a problem? Would you say hardly ever, some of the time, or often?

HARDLY EVER (OR NEVER)

SOME OF THE TIME

OFTEN

DON’T KNOW

REFUSED

**PI5) Emotional support from spouse/partner**

This question was not asked if the respondent did not have a current spouse/partner. When calculating the scale, responses were reverse-coded so that 1 = “often,” 2 = “some of the time,” and 3 = “hardly ever (or never).”

How often can you open up to (NAME) if you need to talk about your worries? Would you say hardly ever, some of the time, or often?

HARDLY EVER (OR NEVER)

SOME OF THE TIME

OFTEN

DON’T KNOW

REFUSED

**PI6) Instrumental support from spouse/partner**

This question was not asked if the respondent did not have a current spouse/partner.

How often can you rely on (NAME) for help if you have a problem? Would you say hardly ever, some of the time, or often? SPRELY

HARDLY EVER (OR NEVER)

SOME OF THE TIME

OFTEN

DON’T KNOW

REFUSED

**PI7) Lack companionship**

This question is one of three items included in the three-item loneliness scale developed by Hughes, et al. (2004). It was asked on the leave-behind questionnaire.

How often do you feel that you lack companionship?

HARDLY EVER (OR NEVER)

SOME OF THE TIME

OFTEN

**PI8) Feel left out**

This question is one of three items included in the three-item loneliness scale developed by Hughes, et al. (2004). It was asked on the leave-behind questionnaire.

How often do you feel left out?

HARDLY EVER (OR NEVER)

SOME OF THE TIME

OFTEN

**PI9) Feel isolated**

This question is one of three items included in the three-item loneliness scale developed by Hughes, et al. (2004). It was asked on the leave-behind questionnaire.

How often do you feel isolated?

HARDLY EVER (OR NEVER)

SOME OF THE TIME

OFTEN

**LUBBEN SOCIAL NETWORK SCALE (LSNS-6, Lubben 2006)**

**FAMILY:** Considering the people to whom you are related by birth, marriage, adoption, etc...

1. How many relatives do you see or hear from at least once a month?
2. How many relatives do you feel at ease with that you can talk about private matters?
3. How many relatives do you feel close to such that you could call on them for help?

**FRIENDSHIPS:** Considering all of your friends including those who live in your neighborhood

1. How many of your friends do you see or hear from at least once a month?
2. How many friends do you feel at ease with that you can talk about private matters?
3. How many friends do you feel close to such that you could call on them for help?

SCALE: 0 = none; 1 = one; 2 = two; 3 = three or four; 4 = five thru eight; 5 = nine or more

**OTHER QUESTIONS AND SCALES**

| How would you describe your current state of physical health in general? Would you describe it as excellent, very good, good, discreet, or poor? | |
| --- | --- |
| 1. Poor |  |
| 2. Discreet |  |
| 3. Good |  |
| 4. Very good |  |
| 5. Excellent |  |

| What about your emotional or mental health? Would you describe it as excellent, very good, good, discreet, or poor? | |
| --- | --- |
| 1. Poor |  |
| 2. Discreet |  |
| 3. Good |  |
| 4. Very good |  |
| 5. Excellent |  |

*How often, during the past week, have you experienced the following moods.*

|  | **Rarely or not at all** | **Occasionally** | **Occasionalmente** | **Most of the time** |
| --- | --- | --- | --- | --- |
| "I didn't feel like eating; my appetite was poor." |  |  |  |  |
| "I felt depressed." |  |  |  |  |
| "I had the impression that everything I did required an effort." |  |  |  |  |
| "Sleep was not enough" |  |  |  |  |
| "I've been happy" |  |  |  |  |
| "People have been rude" |  |  |  |  |
| "I enjoyed life" |  |  |  |  |
| "I felt sad" |  |  |  |  |
| "I got the impression that people hated me." |  |  |  |  |
| "I haven't concluded much" |  |  |  |  |

*Below is a list of common pathologies. Please indicate whether you currently suffer from these disorders. If you do not have a problem, please continue with the next problem. Finally, we ask you to specify all medical conditions that have not been listed via the "other medical conditions" option at the end of the list.*

| **Do you have this problem?** | No (0) | Yes (1) |
| --- | --- | --- |
| Arthritis | N | Y |
| Gastric or peptic ulcer | N | Y |
| Emphysema, bronchitis, or pulmonary disease | N | Y |
| Asthma | N | Y |
| Stroke, blood clot, or brain hemorrhage | N | Y |
| High blood pressure or hypertension | N | Y |
| Diabetes or high blood sugar | N | Y |
| Alzheimer's disease or other forms of dementia | N | Y |
| Cirrhosis, or severe liver failure | N | Y |
| HIV/AIDS | N | Y |
| Leukemia or polycythemia vera | N | Y |
| Lymphoma | N | Y |
| Skin tumors (including melanomas, basal cell carcinoma (BCC) and spinal-cell carcinoma (SCC)) | N | Y |
| Tumor, in addition to skin cancer, leukemia, or lymphoma | N | Y |
| Renal insufficiency | N | Y |
| Thyroid problems | N | Y |
| Other medical disorders |  |  |
| _______________________________________ | N | Y |
| _______________________________________ | N | Y |

| 1. Gender | |
| --- | --- |
| Male |  |
| Female |  |

| 1. Age |
| --- |
| _______ Years |

| Please indicate your marital status: | |
| --- | --- |
| Married |  |
| Relationship of cohabitation |  |
| 1. Widowed |  |
| 1. Separate |  |
| 1. Divorced |  |
| 1. Single (never married) |  |

| 1. Nation of origin | |
| --- | --- |
| 1. Italian (Italy) |  |
| Swiss |  |
| 1. German (Germany) |  |
| 1. French (France) |  |
| 1. Other origin. Please specify: ______________________________ |  |

| What is the last educational level you have achieved? | |
| --- | --- |
| No diploma obtained |  |
| 1. Primary schools diploma |  |
| 1. Middle school diploma |  |
| 1. Diploma or vocational baccalaureate (apprenticeship) |  |
| 1. High school diploma |  |
| 1. University of Applied Sciences diploma |  |
| 1. University degree (three-year, specialist, doctorate) |  |
| 1. Other. Please specify:____________________________ | |
